# Development of an aptamer-based rapid diagnostic assay for *Salmonella* detection using whole-cell SELEX technique

**DOI:** 10.1128/spectrum.03319-25

**Published:** 2026-02-13

**Authors:** Abebe M. Aga, Demise Mulugeta, Abera Motuma, Atsbeha Gebreegziabxier Weldemariam, Adugna Abdi Woldesemayat, Bilise Wakitole, Fanos Tadesse Woldemariyam, Chala Bashea, Musin Kelel, Zinash Teferi, Gemechu Chala Hunderra, Rajiha Abubeker, Gebremedhin Gebremicael, Abraham Ali, Gemechu Tadesse Leta, Natnael Berihun, Shambel Tadesse, Serkadis Oljira, Dereje Nigussie, Mesfin Tafesse Gemeda

**Affiliations:** 1 Armauer Hansen Research Institute, Vaccines, Diagnostics and Medical Device R&D, Addis Ababa, Ethiopia; 2 Department of Biotechnology and Bioprocess Centre of Excellence, Addis Ababa Science and Technology University 446348, Addis Ababa, Ethiopia; 3 Ethiopian Public Health Institute, Integrated Pathogen Genomics and Bioinformatics Facility for Surveillance and Research 128164, Addis Ababa, Ethiopia; University of Arkansas for Medical Sciences, Little Rock, Arkansas, USA

**Keywords:** *Salmonella* detection, DNA aptamer, nanoparticles, colorimetric assay, whole-cell SELEX, point-of-care diagnostics

## Abstract

Rapid and accurate detection of *Salmonella* is essential for improving food safety and reducing the burden of enteric infections, especially in low-resource settings. Aptamer-based diagnostic platforms offer a promising alternative to antibody-based methods, given their high specificity, stability, and low production cost. A DNA aptamer targeting *Salmonella enterica* was developed through Systematic Evolution of Ligands by Exponential Enrichment (SELEX) iterative rounds of selection. Target-binding efficiency was monitored by colorimetric and spectrophotometric binding tests. Silver nanoparticles (AgNPs) were synthesized and conjugated with candidate aptamers through direct adsorption. Dot blot colorimetric assays were performed by exposing aptamer–AgNP complexes to serial dilutions of *Salmonella* cultures ranging from 10² to 10⁸ CFU/mL. Aggregation was evaluated visually on nitrocellulose membranes and quantified by a UV-Vis spectrophotometer based on absorbance intensity and peak wavelength shifts. Controls included AgNPs alone and related non-target bacterial (*Escherichia coli*). Final aptamer pools were sequenced using Nextseq 550 Illumina platform to determine specific sequences and predict candidate secondary structures for future mass production. Aptamer selection showed progressive enrichment, with significant dot blot signal and aggregation from round 8, and strong binding after 12 rounds of SELEX. Upon exposure to *Salmonella*, aptamer–AgNP complexes exhibited a concentration-dependent aggregation response, with visual detection observed at bacterial concentrations of 10^5^ CFU/mL and selective binding toward *Salmonella* compared to *E. coli* and spectrophotometrically quantifiable from 10⁵ CFU/mL. Peak absorbance increased from 0.31 AU for 10² CFU/mL target concentration to 1.73 AU with 10⁸ CFU/mL, and the wavelength shifted from 504 nm to 525 nm, indicating nanoparticle aggregation. No specific aggregation or spectral shifts were observed in control samples. The enriched aptamer candidates were identified and prioritized, exhibiting conserved secondary structures, including hairpins, multi-branched loops, and internal bulges, predicting to facilitate specific interactions with Salmonella outer membrane proteins or LPS-O antigen. This study demonstrates the diagnostic applicability of aptamer conjugated with AgNP colorimetric assays for sensitive and specific detection of *Salmonella*. Future work should focus on sequence-based chemical modifications to enhance aptamer immobilization and assay performance, thereby facilitating adaptation of the platform to lateral flow formats during mass production for point-of-care applications.

**IMPORTANCE:**

This study addresses a critical need for rapid, low-cost, and sensitive diagnostic tools for Salmonella detection, particularly in low-resource settings where conventional methods are often inaccessible or time-consuming. By developing an aptamer-based colorimetric assay using silver nanoparticles, the research offers a highly specific, sensitive, and affordable alternative to existing diagnostics. The use of DNA aptamers selected through Systematic Evolution of Ligands by Exponential Enrichment enables precise recognition of Salmonella surface markers, while the colorimetric readout allows for easy visual detection without the need for sophisticated instruments. The assay’s sensitivity demonstrates its practical utility for early and field-level pathogen detection. Importantly, the incorporation of next-generation sequencing in aptamer characterization enhances the molecular understanding of aptamer–target interactions and supports future assay refinement for application in lateral flow assay. The findings support translating the platform into point-of-care formats, which help food safety monitoring and infectious disease control in developing regions.

## INTRODUCTION

*Salmonella* is a significant pathogenic bacterium responsible for salmonellosis, a major global foodborne disease affecting both humans and animals. Non-typhoidal *Salmonella* alone causes approximately 93.8 million cases and 155,000 deaths annually, with sub-Saharan Africa bearing a disproportionate share of invasive infections, where case-fatality rates can exceed 20% among children and immunocompromised individuals (1). The genus *Salmonella* comprises two species, *Salmonella enterica* and *Salmonella bongori*, with *S. enterica* responsible for human illness and foodborne outbreaks worldwide, particularly due to serovars *Typhimurium* and *Enteritidis* (2). According to the World Health Organization (WHO), unsafe food results in approximately 600 million cases of foodborne diseases and 420,000 deaths each year, with *Salmonella* being a major contributor (3). The burden is disproportionately high in low- and middle-income countries, where inadequate access to clean water, sanitation, diagnostic services, and healthcare systems exacerbates the impact (4, 5).

Traditional diagnostic techniques such as culture-based assays and polymerase chain reaction (PCR) are widely used for *Salmonella* detection and remain the gold standard. However, these methods are time-consuming, labor-intensive, and require specialized infrastructure and trained personnel (6). These constraints limit their utility in outbreak settings and resource-constrained environments. Additionally, the growing threat of antimicrobial-resistant (AMR) *Salmonella* strains complicates treatment efforts and further underscores the urgent need for rapid, accurate, and field-deployable diagnostic tools (7–9). In this context, there is a critical need for simple, rapid, and effective diagnostic methods that can be deployed at the point of care or in field settings. Such tools are essential not only for the timely detection of *Salmonella* infections but also for the surveillance of emerging drug-resistant strains. Early and accurate diagnosis supports prompt clinical decision-making, facilitates outbreak containment, and strengthens public health responses (10, 11). A low-cost, user-friendly, and sensitive platform, such as an aptamer-based rapid test, bridges current diagnostic gaps, offering a practical solution for improving detection and control across both clinical and food safety sectors (12–14). Other studies highlight the powerful role of aptamers in enhancing the sensitivity, specificity, and versatility of dot blot assays for biomolecular detection (15). Aptamer-based biosensors have shown effective use in food matrices and field samples, highlighting their real-world applicability (12, 16). Its simplicity and specificity make it suitable for field applications in clinical, food safety, and environmental surveillance. By enabling early detection, this approach contributes to improve *Salmonella* monitoring and helps reduce unnecessary antibiotic use, supporting AMR control efforts.

Aptamer-based biosensors have emerged as a promising class of diagnostic platforms due to their high specificity, stability, and adaptability. Aptamers are short, single-stranded DNA (ssDNA) or RNA oligonucleotides selected via the Systematic Evolution of Ligands by Exponential Enrichment (SELEX) process (17, 18). They bind specifically and tightly to their targets and offer several advantages over antibodies, including thermal stability, target recognition, chemical flexibility, and cost-effective synthesis. These properties make aptamers especially attractive for point-of-care applications and biosensor development (19, 20).

Studies have demonstrated the effectiveness of aptamer-based detection platforms for *Salmonella*, including electrochemical aptasensors achieving effective detection and fluorescence-based aptasensors utilizing hollow carbon nitride nanospheres for *Salmonella Typhimurium* (11, 21). Additionally, an impedimetric biosensor employing whole-cell SELEX-derived aptamers detected *S. enteritidis* within 10 min (17). Other studies developed a colorimetric aptamer-based sensor using polydiacetylene-liposome conjugates, which produced visible results within 15 min (22, 23). Colorimetric nanoparticles, especially silver and gold, provide strong surface plasmon resonance properties that enable visible color changes upon aggregation, making them powerful signal transducers in aptamer-based biosensors (24). Their large surface-to-volume ratio also allows high-density aptamer immobilization, enhancing both sensitivity and dynamic range in detecting pathogens like *Salmonella* under resource-limited conditions (25).

Recent advancements in aptamer selection using whole-cell SELEX have significantly enhanced the development of rapid diagnostic tools for bacterial pathogens such as *Salmonella*. Unlike traditional methods that rely on purified targets, whole-cell SELEX enables the selection of aptamers that bind specifically to live bacterial cells without prior knowledge of their surface proteins, allowing recognition of intact surface markers in their native conformation. This improves both the specificity and diagnostic performance of aptamer-based assays (26, 27). The secondary structure of aptamers, particularly stem-loops, bulges, and multi-junction motifs, plays a critical role in defining target recognition, as these conformations form unique three-dimensional binding pockets essential for high-affinity interactions with *Salmonella* surface molecules (28). Moreover, stable secondary structures enable efficient functionalization, such as thiol- or amine-modification for nanoparticle conjugation, thereby ensuring both specificity and structural resilience in biosensing platforms (29). These structural and functional sequence folding support the integration of aptamer-based recognition elements into point-of-care testing platforms such as lateral flow assays, offering promising alternatives for sensitive and selective detection of *Salmonella* spp. in complex samples. By enabling the generation of aptamers that recognize the overall surface architecture of the pathogen, this approach is particularly valuable for complex organisms like *Salmonella enterica*, which comprises multiple serovars with diverse antigenic profiles (22, 30, 31). The aim of this study was to develop and validate a rapid, sensitive, and specific aptamer-based diagnostic assay for *Salmonella enterica* detection using whole-cell SELEX coupled with nanoparticle-mediated colorimetric signal transduction. In addition to SELEX enrichment, this work emphasizes rigorous candidate selection by prioritizing sequences based on abundance, conserved structural motifs, and predicted stability to ensure suitability for conjugation, immobilization, and robust performance.

## MATERIALS AND METHODS

### ssDNA generation

Genomic DNA was extracted from confirmed *Salmonella enterica* isolates in mixed serovar culture using the Qiagen DNeasy Blood and Tissue Kit, following the manufacturer’s instructions (Hilden, Germany). Bacterial cells were lysed, and DNA was purified via a spin column extraction protocol. The concentration and purity of the extracted DNA were assessed using a Qubit fluorometer (Thermo Fisher Scientific, USA). The double-stranded DNA (dsDNA) was denatured by heating at 95°C for 10 min, followed by snap cooling on ice to obtain linear ssDNA. To produce short ssDNA fragments suitable for SELEX, genomic DNA was digested separately with EcoRI and HindIII restriction enzymes (New England Biolabs, USA) at 37°C for 1 h to reduce DNA fragment size. The digested products were purified with a Qiagen purification kit.

### Whole-cell SELEX for aptamer screening

The cell-SELEX was conducted over 14 iterative rounds to isolate ssDNA aptamers with high affinity and specificity for a mixed culture of *Salmonella enterica* serovars.

**Fig 1:**
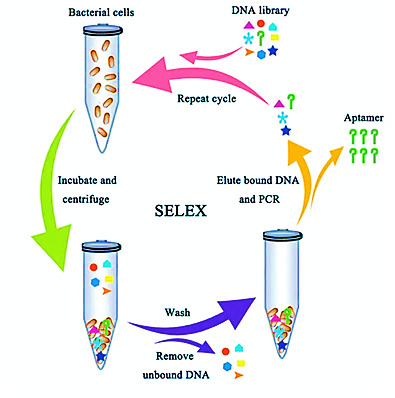
Cell-SELEX process for aptamer selection and screening. To enhance specificity, counter-selection steps were introduced in rounds 1, 3, and 8 by pre-incubating the ssDNA pool with *Escherichia coli* at 37°C for 30 min and discarding sequences that bound to the non-target bacteria. After 12 rounds of SELEX, the enriched DNA pool was processed to generate a standardized ssDNA library suitable for sequencing and diagnostic development. This was achieved through PCR amplification using commercial random primers, yielding fragments with a controlled length distribution of approximately 80–150 base pairs and introducing additional sequence diversity around enriched binding motifs. The final enriched pool was then evaluated for target specificity using binding assays against both *Salmonella* and non-target bacterial species, confirming selective binding of the enriched sequences. Subsequently, the pool was sequenced and analyzed bioinformatically to identify unique sequences, cluster aptamer families, and predict secondary structures, enabling rational candidate selection for downstream diagnostic assay development.

The initial DNA library was generated from extracted genomic DNA of confirmed *Salmonella enterica* isolates. Genomic DNA was enzymatically fragmented to generate short DNA fragments, which were subsequently converted to ssDNA prior to selection. At the beginning of each SELEX round, the DNA pool was heat-denatured at 95°C for 5 min and immediately snap-cooled on ice for 5 min to ensure strand separation. The ssDNA pool was then incubated at room temperature for 10 min to allow refolding into secondary structures before incubation with target bacterial cells. In each SELEX round, 10^6^ CFU/mL of live *Salmonella* whole cells were incubated with the ssDNA library in binding buffer at 37°C for periods ranging from 1 h (early rounds) to 10 min (later rounds), with incubation time decreasing as selection stringency increased. Following incubation with intact bacterial cells, unbound ssDNA sequences were removed through multiple washing steps, and target-bound aptamers were recovered by heat denaturation to dissociate them from the bacterial surface. This denaturation–refolding cycle was applied consistently at each SELEX round to maintain single-strandedness and promote formation of stable, reproducible DNA conformations. Iterative selection under increasing stringency progressively enriched high-affinity target-binding sequences while eliminating non-binding or weakly folded fragments. Washing stringency increased progressively across SELEX cycles to enrich high-affinity binder early rounds featured minimal washing, while later rounds incorporated more rigorous washes to eliminate low-affinity or nonspecific sequences (Fig. 1). Recovered ssDNA from 12th rounds was amplified using commercial random primers to regenerate the library for the next cycles.

### Silver nanoparticle synthesis and aptamer conjugation

Silver nanoparticles (AgNPs) were synthesized using a chemical reduction method from silver nitrate (Sigma-Aldrich, USA), which served as the silver source. A 2 mM solution of silver nitrate was prepared and heated to 80°C with continuous stirring. AgNPs were synthesized by adding citrate as a suitable reducing agent under controlled temperature and pH conditions. The reaction mixture was maintained under heat and stirring for 30 min until a color change from pale yellow to light brown indicated successful AgNP formation. The solution was then cooled to room temperature, purified by centrifugation at 8,000 rpm for 15 min, and the resulting pellet was resuspended in 1 mM trisodium citrate. The colloidal AgNPs were stored at 4°C in a light-protected container until use. To prepare aptamer-AgNP conjugates, selected and screened ssDNA aptamer candidates were heated at 95°C for 5 min and snap-cooled on ice for annealing. To form a 3D structure, the aptamer was left at room temperature for 10 min, and a 1:10 ratio of aptamer to AgNPs was combined and incubated under optimized conditions (pH 7.4). Conjugation was achieved by passive adsorption with a 2-h incubation at room temperature with inverting every 10 min. The mixture was then centrifuged at 8,000 rpm for 10 min to remove unbound aptamers, and the pellet conjugates were resuspended in PBS at pH 7.2. Successful conjugation was confirmed by a shift in absorbance peaks using a UV-1800 spectrophotometer (Shimadzu, Japan).

### Dot blot assay for target binding and limit of detection test

The binding affinity and specificity of the aptamer-AgNP conjugates were assessed using a dot blot assay. *Salmonella enterica* and non-target *E. coli* bacteria were prepared by harvesting cells from overnight cultures in tryptic soy broth, then washing with PBS, and killing bacteria culture by boiling at 95°C for 10 min. Then, 3 μL of each separate killed and live cells was spotted onto nitrocellulose membranes and allowed to air dry at room temperature for 30 min to fix the samples. The membranes were blocked with a solution of 5% polyethylene glycol and 0.1% Tween-20 in PBS for 1 h to reduce nonspecific binding. After blocking, the membranes were spotted with 3 µL of aptamer-AgNP conjugate at each target spot and incubated for 20 min at room temperature. Unbound conjugates were removed by washing the membranes three times with PBS containing 0.05% Tween-20. Specific binding was visualized by the appearance of purple-blue aggregates on the membranes where the aptamer-AgNPs bound to *Salmonella* cells. To determine the limit of detection (LOD), serial dilutions of combined *Salmonella enterica*, ranging from 10⁸ to 10^1^ CFU/mL, were spotted to the membranes as stated previously, and the lowest concentration producing a visible purple-blue aggregation was recorded as the LOD. The designed assay detects the presence of *Salmonella* through specific aptamer–target binding to surface-associated molecular motifs and is independent of bacterial viability. Negative controls included *E. coli* cells and phosphate-buffered saline spots, which were used to assess non-specific binding and background signal.

### Target binding and LOD test using spectrophotometer

The binding and LOD of the aptamer-functionalized AgNP complex for *Salmonella enterica* was evaluated using a UV-1800 spectrophotometer (Shimadzu, Japan). The AgNP–aptamer conjugate was mixed with *S. enterica* at 10-fold serial dilutions ranging from 10⁸ to 10¹ CFU/mL and incubated for 15 min at room temperature. After incubation, 1 mL of each reaction mixture was transferred into a 1 cm path length quartz cuvette. The spectrophotometer was blanked using distilled water before each measurement. Absorbance spectra were recorded over the wavelength range of 300–800 nm, with particular focus on the localized surface plasmon resonance peak shift corresponding to bacterial concentration and binding efficiency. Each sample was scanned at a speed of 400 nm/min with a spectral bandwidth of 1 nm. Both the absorbance intensity at the peak wavelength (λmax) and the corresponding peak wavelength shifts were recorded automatically by the instrument software and analyzed separately as functions of bacterial concentration. Control samples included AgNPs without aptamer conjugation (blank) and aptamer–AgNP conjugates incubated with non-target *Escherichia coli* cells at 10⁶ CFU/mL, which were analyzed under identical conditions for comparison.

### Aptamer amplification, library preparation, and sequencing

Aptamer candidates were further amplified by using commercial random primers with enriched ssDNA sequences. PCR was performed under optimized thermal cycling conditions by initial denaturation at 95°C for 3 min; followed by 20 amplification cycles, denaturation at 95°C for 30 s, annealing at 58°C for 30 s, and extension at 72°C for 30 s; with a final extension step at 72°C for 5 min. The resulting PCR products were analyzed by 2.5% agarose gel electrophoresis to confirm amplification specificity and size. Purified PCR products were quantified by Qubit 1× dsDNA HS Assay Kits (Thermo Fisher Scientific, USA) before library preparation. Library preparation was carried out using the Illumina DNA Prep Kit (Illumina, USA) according to the manufacturer’s recommendation. Following the manufacturer’s guidelines, purified and PCR-amplified single-stranded aptamers were first subjected to tagmentation using bead-linked transposomes and tagmentation buffer 1 (TB1). To enable multiplexed sequencing, unique dual-index adapters were ligated to the tagmented fragments, and library cleanup was performed using Illumina purification beads to selectively retain library sizes suitable for sequencing. The concentration of the final library was assessed using the Qubit dsDNA High Sensitivity Assay Kit (Thermo Fisher Scientific, USA) and then diluted to the appropriate starting concentration for sequencing on the NextSeq 550 platform. The sequencing-ready library was denatured with 0.2 M sodium hydroxide (Illumina) and diluted to the final loading concentration according to the Illumina NextSeq 550 standard protocol. The final diluted library was loaded onto the cartridge, and sequencing was initiated on the flow cell using the automated loading system. After sequencing was completed, the machine generated base call (BCL) files, which were used for downstream analysis. Candidates were ranked by read frequency, enrichment across rounds, predicted secondary structure stability, and motif conservation. Sequencing was conducted at the Integrated Pathogen Genomics and Bioinformatics Facility for Surveillance and Research, Ethiopian Public Health Institute, Addis Ababa, Ethiopia.

### Data analysis

Raw data were imported into SPSS version 25 and cleaned to identify and correct any missing or inconsistent entries. Dot blot and absorbance data were analyzed for LOD signal intensity, and UV-Vis spectrophotometry was used to quantify target binding, enabling evaluation of binding efficiency and assay sensitivity. Results were presented in bar graphs showing mean intensity and absorbance values with standard deviations, and in tables summarizing concentration ranges, aggregation signal responses, and calculated limits of detection. Raw sequence reads from enriched aptamer pools were demultiplexed and converted to FASTQ format. Read quality was assessed with FastQC, and adapters with low-quality bases were trimmed using Trimmomatic. For paired-end data sets, overlapping reads were merged with PEAR to reconstruct full-length aptamer sequences. Processed reads were then screened to remove constant primer-binding regions, and variable aptamer domains were extracted using custom scripts. Unique sequences were identified, and their relative abundances were calculated to determine the most enriched candidates. Secondary structures of the enriched aptamer candidates were predicted using Mfold to support comparative analysis and candidate prioritization. The predicted folding patterns represent computationally derived conformations and are presented as predicted structural arrangements. These predictions were used to identify conserved motifs and assess thermodynamic stability. This evaluates minimum free energy (MFE, kcal/mol), base-pairing patterns, stem lengths, and loop sizes, assessing structural stability. Structural motifs, including hairpins, internal bulges, multi-branched scaffolds, and multi-helix junctions, were documented using dot-bracket notations. Candidate aptamers were mapped against known *Salmonella* surface molecules, including outer membrane proteins, OmpC, OmpF, OmpA, and LPS O-antigen, to identify probable interaction sites. Structural motifs and predicted binding regions were documented to guide the selection of high-affinity aptamers. Aptamer candidates were ranked based on read frequency, predicted structural stability, and motif complexity, highlighting sequences with strong potential for target binding.

## RESULTS

The SELEX process demonstrated a progressive enrichment in aptamer binding efficiency across iterative rounds of selection. Initial rounds of selection (1–3) showed no visible aggregation on the dot blot signal, indicating weak or non-specific binding (Table 1). Beginning at round 8, a low-intensity dot blot signal and mild aggregation were observed, suggesting emerging target specificity. By round 12, strong dot signals and high aggregation levels became apparent. Following PCR amplification, rounds 13–15 consistently produced deep aggregation, indicating enrichment of high-affinity aptamers against *Salmonella*. The detection relied on recognition of *Salmonella*-specific surface molecular signatures, with comparable binding observed for live and heat-inactivated cells.

**TABLE 1:** Aptamer selection and dot blot assay aggregation intensity across SELEX rounds

SELEX round	Dot blot signal	Aggregation intensity
3	No aggregation	None
8	Dot aggregation	Low
12	Dots of aggregation	High
12 + PCR	Aggregation	High
13 + PCR	Deep aggregation	High
14 + PCR	Deep aggregation	High
15 + PCR	Deep aggregation	High

Colorimetric assays employing aptamer-conjugated AgNPs demonstrated a clear concentration-dependent blue aggregation response upon exposure to *Salmonella* (Table 2). Negative controls, including AgNPs alone, AgNP–aptamer conjugates without bacteria, and non-target bacteria (*E. coli*) showed no aggregation. High *Salmonella* concentrations (≥10⁷ CFU/mL) triggered strong aggregation with deep color development, corresponding to a visual intensity score of +5. Moderate aggregation was observed at 10⁵–10⁶ CFU/mL (intensity scores +2 to +4), while faint or no aggregation occurred at lower concentrations (10⁴ CFU/mL), with +1, and at ≤10^3^ no visible aggregation intensity was observed.

**TABLE 2:** Colorimetric method of dot blot assay aggregation response to *Salmonella* and *E. coli*

Sample type	Spot volume (µL)	Bacterial concentration (CFU/mL)	Aggregation interpretation	Visual dot intensity
AgNPs alone	3	0	0	No aggregation
AgNPs_Aptamer	3	0	0	No aggregation
AgNPs_Apt_Salm.	3	1× 10⁸	+5	Deep aggregation
AgNPs_Apt_Salm.	3	1× 10⁷	+5	Deep Aggregation
AgNPs_Apt_Salm.	3	1× 10⁶	+4	Aggregation
AgNPs_Apt_Salm.	3	1× 10⁵	+2	Slightly aggregated
AgNPs_Apt_Salm.	3	1× 10⁴	+1	Faint
AgNPs_Apt_Salm.	3	1× 10³	+1	Very faint
AgNPs_Apt_Salm.	3	1× 10²	0	No aggregation
AgNPs_Apt_E.Coli	3	1× 10⁶	0	No aggregation

**Fig 2:**
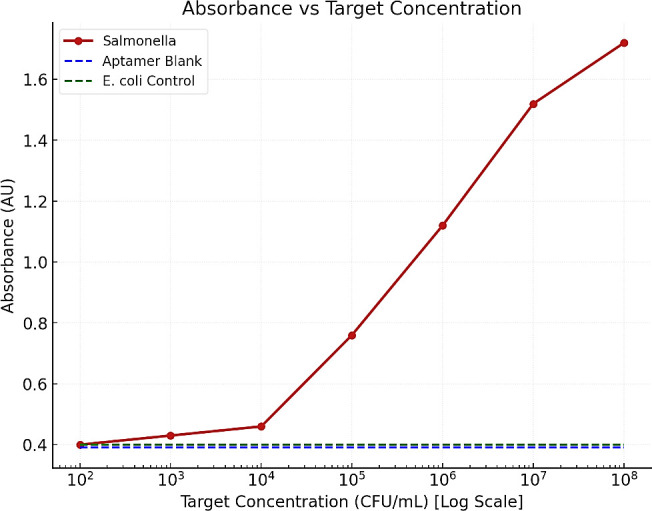
Absorbance response of the aptamer-AgNP conjugation to increasing concentrations of *Salmonella*. Absorbance (AU) is plotted against target concentration (CFU/mL, log scale). The *Salmonella* curve (red line with markers) shows a concentration-dependent increase in signal.

The colorimetric assay showed a direct correlation between absorbance intensity and *Salmonella* concentration (Fig. 2). At low concentrations (10²–10⁴ CFU/mL), absorbance values remained low, ranging from 0.31 to 0.45 AU, with no aggregation of AgNP–aptamer complexes. As the concentration increased, a gradual rise in absorbance was observed: 0.76 AU at 10⁵ CFU/mL, 1.12 AU at 10⁶ CFU/mL, and 1.51 AU at 10⁷ CFU/mL. The maximum absorbance of 1.73 AU occurred at 10⁸ CFU/mL, signifying extensive aggregation due to target binding. These results indicate dose-dependent enhancement in optical density, with increasing levels of nanoparticle aggregation and visible color change.

**Fig 3:**
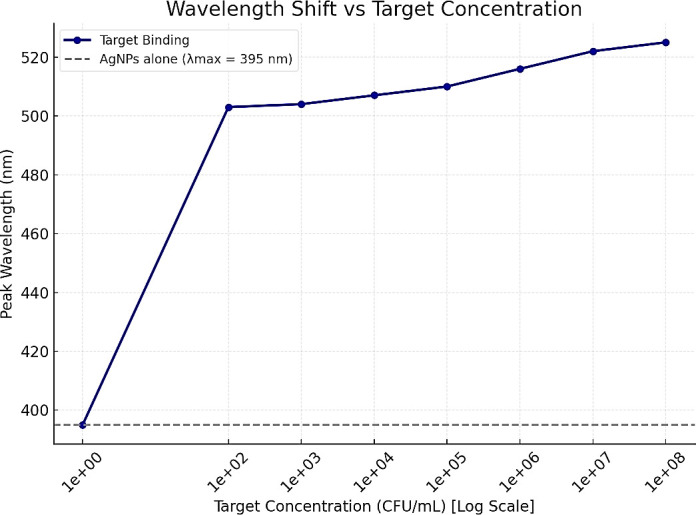
Wavelength (nm) vs target concentration (CFU/mL) for aptamer target binding analysis. The graph illustrates aggregation intensity and absorbance peak wavelength with increasing target concentration, demonstrating the specificity and sensitivity of the aptamer-functionalized AgNPs.

The peak wavelength of the AgNP–aptamer conjugate exhibited a shift as the concentration of *Salmonella* increased (Fig. 3). At low bacterial loads (10²–10⁴ CFU/mL), the peak wavelength remained close to 504–507 nm, consistent with minimal nanoparticle aggregation. As the concentration rose to 10⁵ CFU/mL and above, the wavelength progressively shifted toward longer values: 510 nm at 10⁵ CFU/mL, 516 nm at 10⁶ CFU/mL, 521 nm at 10⁷ CFU/mL, and peaking at 525 nm for 10⁸ CFU/mL. This red shift in surface plasmon resonance indicates increased inter-particle interactions and aggregation, validating the aptamer’s binding-induced colorimetric response. A distinct red shift in the absorbance peak wavelength was observed with increasing *Salmonella* concentration. The maximum absorbance peak shifted from 501 nm at 10² CFU/mL to 525 nm at 10⁸ CFU/mL, indicating nanoparticle aggregation due to target binding. Control conditions (AgNPs alone or with *E. coli*) maintained shorter peak wavelengths around 407–412 nm, typical of non-aggregated nanoparticles. The shift in peak wavelength provides evidence of successful aptamer-mediated detection and could be used as a spectral signature for qualitative analysis in colorimetric biosensors. These functional results, combined with sequencing analysis, enabled us to transition from bulk pool binding to prioritization of individual aptamer candidates for future LFA adaptation.

Sensitivity evaluation using dot blot and spectrophotometric assays demonstrated consistent and reproducible signal discrimination at *Salmonella* concentrations ranging from 10⁵ CFU/mL. Although signal development was occasionally observed at 10³ CFU/mL, the limited sample volume applied (3 µL per dot) resulted in a very low absolute number of bacterial cells, producing inconsistent signals near background levels. Therefore, 10⁴ CFU/mL was defined as the practical lower limit of reliable detection under the current assay conditions.

Sequencing of enriched aptamer pools after 12 rounds of SELEX and three additional consecutive enrichment yielded multiple high-frequency candidate sequences (Table 3). Apt-1 (148 nt) was identified 10 times, representing 22.2% of the top sequences. It exhibited the lowest MFE (–59.8 kcal/mol), indicating strong thermodynamic stability. Predicted secondary structure analysis revealed stacked stem-loops with hairpins, with a maximum stem length of 8 bp and loop size of 8 nt. Stability scoring was 0.933, and binding score was 0.889. *In silico* mapping suggested binding to conserved outer membrane proteins (OmpC, OmpF, and OmpA) and LPS O-antigen mainly through electrostatic, hydrogen bonding, and van der Waals interactions. Apt-2 (149 nt) occurred nine times, corresponding to 20% of the pool. The predicted secondary structure was a multi-branch junction with central stem-loops, with a maximum stem length of 11 bp and a loop size of 7 nt. Its MFE was –47.4 kcal/mol. Stability scoring was 0.671, with a binding score of 0.798. The predicted binding region was lipopolysaccharide O-antigen polysaccharides. Apt-3 (149 nt) was the most frequent sequence, detected 11 times (24.4%). The predicted structure included hairpin stem-loops with internal bulges, with a maximum stem length of 13 bp and loop size of 5 nt. The MFE was –47.9 kcal/mol with stability scoring 0.629 and a binding score of 0.742. Predicted binding regions include OMPs (OmpC, OmpF, and OmpA). Like Apt-1, Apt-4 (149 nt) appeared nine times (20%) and its predicted structure was characterized by internal bulges and side hairpins, with a maximum stem of 11 bp and loop size of 6 nt. The MFE was –49.9 kcal/mol with stability scoring 0.653 and binding score 0.699. Predicted binding regions included LPS O-antigen polysaccharides. The top four aptamer candidates accounted for over 86% of the enriched pool. They exhibited diverse structural motifs, favorable MFEs, and stability and binding scores ranging from 0.629 to 0.933 and 0.699 to 0.889, respectively. Predicted target regions included both outer membrane proteins and LPS O-antigen.

**TABLE 3:** Characteristics of representative aptamer candidates identified and selected from sequence pool^*a*^

ID	Frequency (nucleotide length)	MFE (kcal/mol)	Max. stem	Max. loop	Binding score (0-1)	Stability score (0-1)	Secondary structure	Predicted binding region
Apt-1	10 (148)	−59.8	8	8	0.889	0.933	Stacked stem-loops with hairpins	OMPs; OmpC, OmpF, OmpA; LPS O-antigen
Apt-2	9 (149)	−47.4	11	7	0.798	0.671	Stem-loops with central multi-junction	LPS O-antigen polysaccharides
Apt-3	11 (149)	−47.9	13	5	0.742	0.629	Hairpin stem-loops + internal bulges.	OMPs; OmpC, OmpF, OmpA
Apt-4	9 (149)	−49.9	11	6	0.699	0.653	internal bulges and side hairpins	LPS O-antigen polysaccharides

^*a*^ The table summarizes nucleotide length, MFE, structural motifs, predicted binding scores, stability scores, and potential binding regions for the four most frequent aptamer sequences identified after SELEX.

**Fig 4:**
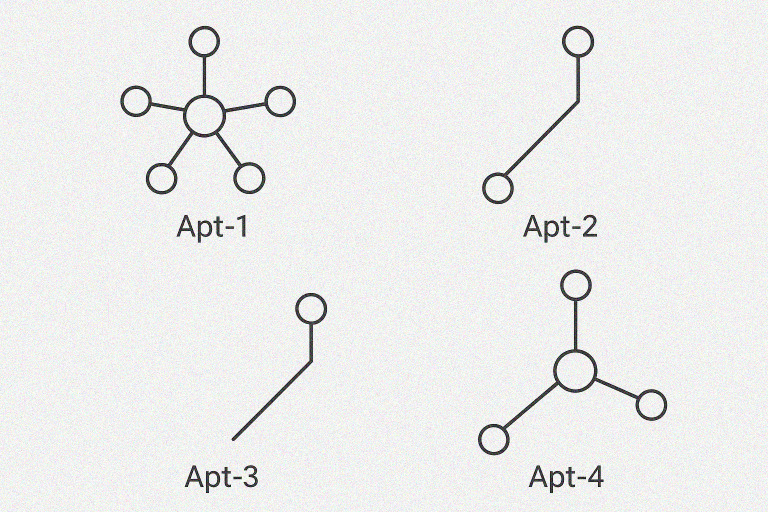
Secondary structure predictions for the top four selected aptamer sequence candidates. Secondary structure predictions generated by Mfold showing multi-branched (Apt-1, Apt-4), extended stem-loop (Apt-2), and linear scaffold (Apt-3) conformations. Each structure shows distinct folding patterns with multiple stems, loops, and branching motifs that contribute to thermodynamic stability and potential target recognition.

Computational secondary structure prediction of the four candidate aptamers displays diverse folding patterns and stem-loop arrangements (Fig. 4). Apt-1 exhibits a star-like structure with multiple short stems radiating from a central loop, suggesting multiple potential interaction sites. Apt-2 shows a more elongated, branched conformation with fewer but longer stems, which may confer stability but limit accessibility. Apt-3 adopts a largely linear, extended structure with small hairpins along its length, potentially allowing flexible binding to target molecules. Apt-4 forms a prominent central loop with three short stems extending outward, creating a defined pocket-like structure that could facilitate specific target recognition. The structural variations observed with variability in structural complexity and potential binding interfaces, and differences in stem length, loop size, and branching are critical factors influencing aptamer-target binding efficiency and stability.

## DISCUSSION

This study demonstrates the successful selection, enrichment, and characterization of *Salmonella*-specific DNA aptamers through a systematic whole-cell SELEX process and their application in a colorimetric detection assay. The progressive enrichment of high-affinity aptamers was evidenced by a clear transition from non-specific responses in early SELEX rounds (1–7) to increasingly specific and intense aggregation signals in later rounds (8–15). Particularly, after round 12 and subsequent PCR amplification, high and consistent aggregation intensities were observed, indicating the successful isolation of aptamers with strong binding affinity to the *Salmonella* target. The result aligns with previous reports, which indicate improved binding efficiency in later SELEX cycles during the development of aptamers against the *Borrelia* surface protein CspZ (32). Similarly, another study demonstrated how hybrid-SELEX led to the enrichment of highly specific aptamers against B7H3, with late rounds showing more stable target interaction and consistent binding (33).

Colorimetric detection using AgNPs conjugated with the selected aptamers proved effective signal generation, exhibiting a pronounced concentration-dependent aggregation response of *Salmonella*. High bacterial concentrations (≥10⁷ CFU/mL) produced deep visual aggregation and strong absorbance responses, while moderate concentrations (10⁵–10⁶ CFU/mL) led to partial aggregation. At low concentrations (≤10⁴ CFU/mL), only faint aggregation was observed, and at ≤10^3^ CFU/mL, no aggregation. The negative controls (AgNPs alone, AgNP–aptamer without target, and non-target *E. coli*) showed no visible aggregation, confirming the aptamer’s specificity and sensitivity in distinguishing *Salmonella* from other bacterial species.

Spectrophotometric analysis further validated the assay’s performance, showing an increase in optical density (absorbance) and wavelength corresponding to increasing *Salmonella* concentrations. Peak absorbance values increased from 0.31 AU at 10² CFU/mL to 1.73 AU at 10⁸ CFU/mL, with a corresponding red shift in peak wavelength from 501 to 525 nm. This shift, a characteristic of nanoparticle aggregation due to target-induced cross-linking, is consistent with other aptamer-based biosensor studies (12, 34). The peak shift observed also reflects similar findings, which described the use of aptamer-mediated aggregation and dot blot assays as a robust method for visually and spectrophotometrically detecting pathogen targets (15). Moreover, the minimal peak shift to related non-target *E. coli* strengthens the case for aptamer selectivity. A similar study emphasized the importance of sequence stringency during SELEX to ensure minimal cross-reactivity in aptamer diagnostics (35). The specificity observed in this study indicates that aptamer–nanoparticle conjugates could serve as a reliable alternative to antibody-based detection methods, as demonstrated by their strong selective response to *Salmonella* at concentrations as low as 10² CFU/mL while showing negligible cross-reactivity with *E. coli*. This finding supports the study, which indicates the potential role of aptamer-based approaches in next-generation diagnostics (12, 35). The SELEX process incorporated multiple rounds of counter-selection using non-target bacteria (*E. coli*) to eliminate non-specific binders, ensuring that the selected aptamers exhibit high specificity for *Salmonella enterica*. While these results indicate target preference, broader specificity assessment against additional non-target bacterial species was not performed and therefore comprehensive exclusivity cannot be concluded.

The integration of dot blot visual recording and spectrophotometric confirmation provides dual-mode validation, increasing assay reliability. The ability to identify target from non-target bacteria visually and spectroscopically suggests strong potential for these aptamers in rapid, field-deployable diagnostic assays. As indicated by a similar study, the final aptamer candidates demonstrated no observable binding to non-target strains during cross-reactivity testing, confirming the specificity of the sequences identified through SELEX (36).

The top four aptamers’ sequences selected accounted for over 86% of the enriched pool after 12 rounds of SELEX, displaying diverse but conserved secondary structures (stem-loops, hairpins, and internal bulges) with MFEs ranging from –47.4 to –59.8 kcal/mol, stability scores of 0.629–0.933, and binding scores of 0.699–0.889. Apt-1, which exhibited the highest frequency and most favorable thermodynamic stability, was predicted to target OMPs (OmpC, OmpF, OmpA) and LPS O-antigen as multiple binding sites, while Apt-2 and Apt-4 were predicted to bind LPS O-antigen motifs, and Apt-3 overlapped with Apt-1 in OMP recognition. Although these targets are conserved among related Enterobacterales species, which could theoretically allow cross-reactivity, the specific three-dimensional folding and local epitope presentation in Salmonella promote selective binding. This division between OMP- and LPS-binding supports prior Salmonella aptamer studies, where aptamers frequently select for outer membrane proteins or polysaccharide epitopes as dominant binding classes. Comparable structural motifs (hairpins, multi-junction loops) and target assignments have been reported in successful cell-SELEX campaigns for gram-negative bacteria, supporting these sequences as strong candidates for broad-spectrum and serovar-specific Salmonella diagnostics (37, 38). Further improvements may include sequence-based structural modifications, such as thiol or biotin incorporation, to enhance stability and strengthen target interactions. Future work should consider aptamer sequence refinement and multivalent use to improve specificity and minimize non-specific binding. Additionally, integration into lateral flow assay platforms, coupled with mass production and field testing, will be essential to enhance applicability at the point-of-care setting.

### Conclusion

This study successfully identified and validated *Salmonella*-specific DNA aptamers through a stepwise whole-cell SELEX process, with functional evaluation using dot blot assays, colorimetric aggregation, and spectrophotometric analysis. The aptamer–AgNP conjugates showed high specificity, producing strong aggregation responses exclusively in the presence of *Salmonella* at concentrations as low as 10⁴ CFU/mL, with no detectable cross-reactivity observed with the non-target bacterium *E. coli*. Quantitative absorbance measurements and wavelength shifts further confirmed the dose-dependent aggregation, providing dual analytical and visual confirmation of detection. Computational structural characterization revealed that the top enriched aptamers predominantly formed hairpin and multi-junction loop motifs, consistent with high-affinity target recognition and supporting their utility for both broad-spectrum and serovar-specific *Salmonella* detection. Leveraging these structural features, the developed assay enables reliable presence-based detection of *Salmonella* through aptamer–target binding irrespective of bacterial viability. Collectively, these findings validate the platform as a promising, low-cost, and rapid alternative to antibody-based diagnostics, with strong potential for point-of-care application in resource-limited settings. Based on these findings, future work should focus on sequence-based structural modifications, such as thiol incorporation to enhance aptamer stability, strengthen aptamer–nanoparticle conjugation, and consistent color development during mass production of lateral flow assay formats. In addition, integration into lateral flow platforms and validation under field conditions are recommended for point-of-care applications in resource-limited settings.

### Study limitations

While this study demonstrates the potential of an aptamer-based rapid diagnostic assay for *Salmonella* detection, certain limitations should be acknowledged. The assay employs unmodified aptamers directly adsorbed onto AgNPs via van der Waals interactions, which may affect binding stability and immobilization efficiency, indicating sequence-level chemical modification is required for improved performance. Specificity evaluation was restricted to only *E. coli* as a non-target organism, and broader assessment against other related enteric bacteria may be required at field conditions. In addition, aptamer secondary structure predictions were based on computational modeling, and after chemical modification, experimental validation of secondary structure may be required.

## Data Availability

The data sets generated and analyzed during this study are available from the corresponding author upon reasonable request. Due to product-related intellectual property protection, full aptamer sequences are not publicly disclosed. Partial sequence data and related analytical details can be provided upon request and may require a confidentiality statement.
